# Concerns regarding hepatitis B vaccination and post-vaccination test among Brazilian dentists

**DOI:** 10.1186/1743-422X-7-154

**Published:** 2010-07-13

**Authors:** Vera Lúcia S Resende, Mauro Henrique G Abreu, Saul M Paiva, Rosângela Teixeira, Isabela A Pordeus

**Affiliations:** 1 Department of Operative Dentistry, Dental School, Federal University of Minas Gerais, Belo Horizonte, Brazil; 2 Department of Community and Preventive Dentistry, Dental School, Federal University of Minas Gerais, Belo Horizonte, Brazil; 3 Department of Paediatric Dentistry and Orthodontics, Dental School, Federal University of Minas Gerais, Belo Horizonte, Brazil; 4 Department of Internal Medicine, Medical School, Federal University of Minas Gerais, Belo Horizonte, Brazil

## Abstract

**Background:**

Hepatitis B infection is the major cause of acute and chronic liver disease, cirrhosis and hepatocellular carcinoma worldwide and has long been recognized as an occupational hazard among dentists. The aim of the present study was to examine factors associated to the self-reporting of hepatitis B vaccination and immunization status among dentists working in the city of Belo Horizonte, Brazil.

**Methods:**

A cross-sectional survey was carried out with 1302 dentists in Belo Horizonte, Brazil. After signing a term of informed consent, the participants answered a structured questionnaire on their knowledge regarding their vaccination and immunization status against hepatitis B. Data on demographic, behavioural and occupational exposure aspects were also collected through questionnaires.

**Results:**

The results revealed that 73.8% of the dentists reported having received three doses of the vaccine. Multivariate analysis revealed that gender (p = 0.006), use of individual protective equipment (p = 0.021), history of blood transfusion (p = 0.024) and history of illicit drug use (p = 0.013) were independently associated with vaccination against hepatitis B. Only 14.8% had performed a post-vaccination test. The use of individual protective equipment (p = 0.038), dentists who asked patients about hepatitis during dental treatment (p < 0.001), a family history of hepatitis B (p = 0.003) and work experience (p < 0.05) were independently associated with the post-vaccination test.

**Conclusions:**

Although there were a large number of vaccinated dentists in Belo Horizonte, the percentage was less than what was expected, as Brazil offers the National Program of Viral Hepatitis Vaccination, which provides free hepatitis B vaccinations to all healthcare workers. Despite being part of a high risk group for contamination, most of the dentists did not know their immunization status.

## Background

Hepatitis B virus (HBV) is the major cause of acute and chronic liver disease, cirrhosis and hepatocellular carcinoma worldwide and has long been recognized as an occupational hazard among dentists [1-6]. A third of the world population (two billion people) has evidence of hepatitis exposure and an estimated 400 million are actively infected [3,7]. HBV is one of the major diseases of mankind and is a serious global public health problem [8,9]. HBV is transmitted primarily through parenteral and sexual exposure to HBsAg-positive blood or other body fluids from individuals who are chronic HBV carriers or have acute hepatitis B [2]. These chronically infected individuals are at high risk of death from cirrhosis of the liver and liver cancer, diseases that kill about one million people each year [10,11].

As the dental profession involves the use of small, sharp instruments contaminated with blood or other fluids, there is ample opportunity for inadvertent skin wounds to the operator and staff [1,2,12]. Such accidents include the possibility of transmission of hepatitis B, hepatitis C and human immunodeficiency virus (HIV) [13]. To decrease the risk of HBV infection, it is recommended that dental personnel receive immunization against HBV and use individual protective equipment, such as gloves, to prevent blood-borne infection during dental procedures [1,11].

The hepatitis B vaccine has been available since 1982 and, since 1990, has been recommended for healthcare workers whose activities frequently expose them to blood [7,10,11,14]. However, 5 to 10% of normal subjects do not produce the anti-hepatitis B surface antibody (anti-HBs) after receiving a standard course of HBV vaccine [7,15,16]. Thus, post-vaccination testing one to three months following the third dose of vaccine is recommended for healthcare workers who have contact with blood [7]. Previous studies carried out in other countries have revealed different proportions of self-reported vaccination, ranging from 40.3% to 97.0% [17-23]. The proportion of dentists who have had their antibody titer evaluated ranges from 36.5% to 47.9% [20,21]. In Brazil, one study found that only 73.1% of dentists had been submitted to the three doses of the vaccine [24].

There is little information on factors associated to adherence to the vaccination and the evaluation of immunization status regarding hepatitis B among dentists. The aim of the present study was to examine factors associated to the self-reporting of hepatitis B vaccination and immunization status among dentists working in the city of Belo Horizonte, Brazil.

## Methods

This is a cross-sectional study nested in a larger study assessing factors associated to the seroprevalence of hepatitis C among dentists in the city of Belo Horizonte, Brazil. The dentists were first contacted, enrolled and invited to take part in the study in November 2004, when all dentists registered at the Minas Gerais Dental Council were required to elect the administrative board of the council. Belo Horizonte is the capital of the state of Minas Gerais and is an industrialized city with about 2.4 million inhabitants. The sample size was calculated to give a 95% confidence interval, 0.75% precision level [25] and using a 3% prevalence of hepatitis C [10]. The inclusion criteria were dentists living and working in the city of Belo Horizonte and registered at the Minas Gerais Dental Council. This group consists of a finite population of 2766 dentists. The minimal sample size to satisfy the requirements was estimated as 1156 dentists. Taking into account the possibility of losses, a correction factor of 1.2 was adopted, totalling an expected sample size of 1387 dentists. These dentists scheduled a visit at the clinical analysis laboratory of the Medical School of the Federal University of Minas Gerais. A total sample of 1302 dentists (response rate = 93.9%) answered the questionnaire between December 2004 and June 2006. The sampling strategy is detailed in a previous article [26]. All participants signed a term of informed consent and data were collected on demographic, behavioural and occupational exposure aspects.

### Questionnaire

A self-administered questionnaire consisting of twenty open-ended and close-ended items was used for the data collection. The drafting of this questionnaire complied with all steps proposed by previous studies [27,28]. Once the purpose of the study and its conceptual basis were defined, the drafting of the items was carried out by means of a broad-based review of the literature [29-32]. Content validation was performed to determine the suitability of the theoretical content and functionality of the questionnaire. Item selection, adaptation and additional inclusions were then performed based on the opinion of an expert in research and marketing. An opinion was subsequently formed by a commission made up of professionals from different dental institutions and specialties. Unanimity in the approval of the questionnaire was required for validation. Suggestions for changes were heeded when brought up repeatedly by different commission members. Response options were organized vertically. All survey items were constructed in the same format in order to avoid placing emphasis on any specific item [26].

In the present study, the following variables were taken into account:

#### 1- Personal and behavioural data

Gender; sexual behaviour (unprotected homo/hetero sex with a casual partner); blood transfusion; previous history of hepatitis in participant or family member; and use of illicit injection drugs. Since the latter variable is quite sensitive, this point was addressed in combination with exposure factors, such as the use of piercing and tattoos, history of any kind of transplant, dialysis, colonoscopy and chemotherapy.

#### 2- Professional, behavioural and occupational exposure data

Work experience; workplace; field of work; use of individual protective equipment (IPE); vaccination for hepatitis B; immunization against hepatitis B; history and number of needle stick accidents with visible bleeding; dental assistance for patients with hepatitis; and whether the dentists' clinical dental chart contains a question on a history of hepatitis.

As individuals may go to school at any time in their lives and the number of years of professional activity may be more important than age in representing exposure to risk factors, the age of the participants was not inquired, but rather the duration of activity (work experience). To analyze the influence of work experience, the sample was categorized into four groups: less than 10 years; 10 to 20 years; 21 to 30 years; and 30 years or more. The workplace was considered public, private or both.

Data were analyzed using the Statistical Package for the Social Sciences (SPSS for Windows, version 17.0, SPSS Inc, Chicago, IL, USA). Bivariate analysis was the initial analytic strategy (Fisher's exact test and Pearson's chi-square test). Multivariate Poisson regression with robust variance was then performed. Vaccination for hepatitis B and immunization against hepatitis B were the dependent variables. The level of significance was set at α = 0.05.

## Results

Among the total number of dentists surveyed, 87.1% (n = 1134) answered the question on hepatitis B vaccination and 74.8% (n = 974) answered the question on immunization for hepatitis B. Among the study sample (n = 1302), 73.9% reported having received the three doses of the vaccine. There was an association between vaccination against hepatitis B and gender (p = 0.003), use of IPE (p = 0.001), blood transfusion (p = 0.006) and combined factors (p = 0.026) in the univariate analysis (Table 1). The multivariate analysis revealed that women had a 1.06-fold (95%CI: 1.02-1.10) greater frequency of vaccination against hepatitis B than men. Dentists who reported the use of IPE had a 1.05-fold (95%CI: 1.01-1.09) greater prevalence of vaccination. Dentists who reported having received no blood transfusions had a 1.10-fold (95%CI: 1.01-1.21) greater prevalence of vaccination. Dentists reported having no combined risk factors had a 1.08-fold (95%CI: 1.02-1.15) greater prevalence of vaccination (Table 2).

**Table 1:** Factors associated with self-reporting of hepatitis B vaccination among Brazilian dentists, Belo Horizonte, 2005

	Hepatitis B vaccination			p-value
	Yes	No	Total	
Gender				
Male	288	71	359	0.003*
Female	674	101	775	
Total	962	172	1134	

Work experience††				
Less than 10 years	264	42	306	0.431*
10 - 20 years	256	41	297	
21 - 30 years	319	51	370	
More than 30 years	115	27	142	
Total	954	161	1115	

Workplace††				
Public	202	34	236	0.945*
Private	408	72	480	
Both	274	50	324	
Total	884	156	1040	

Field ††				
General dentistry	409	52	461	0.067**
Oral Surgery/Periodontology	109	20	129	
Operative Dentistry/Endodontics	131	27	158	
Paediatric Dentistry/Orthodontics	112	26	138	
Oral Public Health	38	4	42	
Oral Pathology/Oral Radiology	18	6	24	
Total	817	135	952	

Use of IPE				
Yes	631	91	722	0.001*
No	331	81	412	
Total	962	172	1134	

Needle stick accidents††				
Yes	829	139	968	0.065*
No	128	32	160	
Total	957	171	1294	

Dental care for hepatitis patients ††				
Yes	264	44	308	0.928*
No	489	83	572	
Total	753	127	880	

Question on patient history of hepatitis ††				
Yes	799	132	931	0.087*
No	153	36	189	
Total	952	168	1120	

Unprotected homo/hetero sex††				
Yes	202	37	239	0.862*
No	752	133	885	
Total	954	170	1124	

Blood transfusion††				
Yes	42	16	58	0.006*
No	881	147	1028	
Total	923	163	1086	

Previous history of hepatitis††				
Yes	100	15	115	0.529*
No	816	147	963	
Total	916	162	1078	

Family history of hepatitis ††				
Yes	227	34	261	0.387*
No	521	94	615	
Total	748	128	876	

Combined question†, ††				
Yes	106	29	135	0.026*
No	829	137	966	
Total	935	166	1101	

* Pearson's chi-square test ** Fisher's exact test

† This question included use of illicit injection drugs, piercings and tattoos, blood transfusion, transplant, history of dialysis, colonoscopy or chemotherapy.

††: For these variables, the sum of the data does not result in 100%, as these questions were not answered by the entire sample.

**Table 2:** Adjusted factors associated with self-reporting of hepatitis B vaccination among Brazilian dentists, Belo Horizonte, 2005

	Prevalence Ratio (95%CI)	p-value
Gender		
Female	1.06 (1.02-1.10)	0.006
Male	1	

Use of IPE		
Yes	1.05 (1.01-1.09)	0.021
No	1	

Blood transfusion		
No	1.10 (1.01-1.21)	0.024
Yes	1	

Combined question		
No	1.08 (1.02-1.15)	0.013
Yes	1	

When asked whether they knew their immunization status or had performed a post-vaccination test, 14.8% of the total sample reported having taken the test. Immunization status was associated with work experience (p = 0.003), the use of IPE (p = 0.003), dental care for patients with hepatitis (p = 0.011), the custom of asking patients about hepatitis during dental treatment (p = 0.001) and a family history of hepatitis (p = 0.004) (Table 3). The multivariate analysis revealed that dentists who reporting the use of IPE had a 1.03-fold (95%CI: 1.00-1.07) greater prevalence of vaccination. Dentists who asked patients about hepatitis had a 1.07-fold (95%CI: 1.04-1.11) greater prevalence of immunization. Dentists who reported a family history of hepatitis had a 1.06-fold (95%CI: 1.02-1.12) greater prevalence of immunization. Dentists with less than 10 years of experience had a 1.07-fold (95%CI: 1.02-1.12) greater prevalence of immunization when compared to dentists with more than 30 years of experience. Dentists with 10 to 20 years of experience had a 1.07-fold (95%CI: 1.02-1.13) greater prevalence of immunization when compared to dentists with more than 30 years of experience. There was no difference in the prevalence of immunization between those with 20 to 30 years of experience and those with more than 30 years of experience (Table 4).

**Table 3:** Factors associated with self-reporting of hepatitis B post-vaccination test among Brazilian dentists, Belo Horizonte, 2005

	Knowledge on immunization status			p-value
	Yes	No	Total	
Gender				
Male	55	256	311	0.253*
Female	138	525	663	
Total	193	781	974	

Work experience††				
Less than 10 years	65	222	287	0.003*
10 - 20 years	62	182	244	
21 - 30 years	55	264	319	
More than 30 years	11	99	110	
Total	193	767	960	

Workplace††				
Public	45	155	200	0.511*
Private	76	332	408	
Both	58	221	279	
Total	179	708	887	

Field††				
General dentistry	72	328	400	0.085**
Oral Surgery/Periodontology	35	87	122	
Operative Dentistry/Endodontics	21	104	125	
Paediatric Dentistry/Orthodontics	27	98	125	
Oral Public Health	4	26	30	
Oral Pathology/Oral Radiology	5	11	16	
Total	164	654	818	

Use of IPE				
Yes	142	484	626	0.003
No	51	297	348	
Total	193	781	974	

Needle stick accidents††				
Yes	171	663	834	0.227
No	22	115	137	
Total	193	778	971	

Dental care for hepatitis patients ††				
Yes	69	199	268	0.011
No	89	406	495	
Total	158	605	763	

Question on patient history of hepatitis ††				
Yes	176	626	802	0.001
No	17	144	161	
Total	193	770	962	

Unprotected homo/hetero sex††				
Yes	37	157	194	0.790
No	154	619	773	
Total	191	776	967	

Blood transfusion††				
Yes	7	40	47	0.393
No	179	717	896	
Total	186	757	943	

Previous history of hepatitis††				
Yes	23	77	100	0.459
No	164	662	826	
Total	187	739	926	

Family history of hepatitis ††				
Yes	61	164	225	0.004
No	96	444	540	
Total	157	608	765	

Combined question†, ††				
Yes	26	107	188	0.935
No	162	654	761	
Total	188	816	949	

* Pearson's chi-square test ** Fisher's exact test

† This question included use of illicit injection drugs, piercings and tattoos, blood transfusion, transplant, history of dialysis, colonoscopy or chemotherapy.

††: For these variables the sum of the data does not result in 100% as these questions were not answered by the entire sample.

**Table 4:** Adjusted factors associated with self-relating of hepatitis B post-vaccination test among Brazilian dentists, Belo Horizonte, 2005

	Prevalence Ratio (95%CI)	p-value
Use of IPE		
Yes	1.03 (1.0-1.07)	0.038
No	1	

Question on patient history of hepatitis		
Yes	1.07 (1.04-1.11)	< 0.001
No	1	

Family history of hepatitis		
Yes	1.06 (1.02-1.10)	0.003
No	1	

Work experience		
Less than 10 years	1.07 (1.02-1.12)	0.004
10 - 20 years	1.07 (1.02-1.13)	0.006
21 - 30 years	1.02 (0.98-1.17)	0.262
More than 30 years	1	

## Discussion

Prevention is ultimately the most efficient and humane means toward improved health [33]. Immunization programs are highly effective, clearly protect populations and individuals at risk and are leading to the elimination of hepatitis B [34]. Viral hepatitis is preventable with effective vaccines, which have been available since 1982 and have proven safe to both adults and children [7,10]. However, despite being safe, efficacious and cost-effective, hepatitis B vaccination remains consistently under-employed [34].

Reports from different countries reveal that some dentists do not engage in safe practices, such as the use of gloves, facemasks or protective eyeglasses. Moreover, hepatitis B virus vaccination coverage is not complete among dentists, as reported for countries such as Nigeria, Jordan, Iran and the United Kingdom [17-23].

The results of the present study reveal that an average of 73.8% of the dentists had taken the three doses of the hepatitis B vaccine. The Brazilian Ministry of Health offers the National Viral Hepatitis Program, which has provided free vaccinations for newborn children, adolescents, those who work in the sex industry and healthcare workers since 1998. Thus, a higher number of vaccinated dentists was expected. However, this is a good coverage compared to countries that do not have government vaccination programs, such as Kenya, where only 12.8% of healthcare workers have been vaccinated for hepatitis B [23]. It is important to point out the differences between the two studies: The present study was carried out in a large city, whereas the Kenyan study was carried out in Thika, which is a typical small Kenyan district [23]. The number of vaccinated dentists in the present study was also higher than that reported for correctional healthcare workers in three American states, in which only 64% of the 411 professionals interviewed reported having received all three doses of the vaccine [35]. On the other hand, vaccination coverage among English dentists is quite higher than that reported in the present study [22].

Female dentists, dentists who use IPE and those who reported the use of illicit drugs had a greater prevalence of vaccination for hepatitis B. A study carried out in the city of Recife (Brazil) found that women adhere to infection control measures more than male dentists [36]. It is troubling that dentists who use illicit drugs and those with a history of blood transfusion have a lower proportion of adherence to the vaccination than those who do not report these factors, considering the increased risk [2]. On the other hand, it appears that the dentists who adhere more to infection control measures, such as the use of EPI, also have a higher prevalence of vaccination. This finding is similar to that reported in a study carried out in Iran [20].

Regarding vaccination status, there were no differences in relation to work experience. This may be explained by the fact that all the three dental schools in the city of Belo Horizonte offer an immunization program for their students before initiating dental practice, which explains why most of the dentists are vaccinated, especially those recently graduated. Those with a longer-standing profession may have been vaccinated during a campaign by the Dental Council of Minas Gerais in 1996, when all dentists in the state were offered free vaccinations. However, vaccination goes beyond taking vaccines; it implies a monitoring process, which is seldom considered including all vaccine series and over the lifetimes of individuals [33].

There was a very low prevalence of hepatitis B immunization. Studies carried out in Iran report that dentists take the test for the evaluation of immunization status with a frequency least 2.5-fold greater than Brazilian dentists [20,21]. This finding underscores the need for collective actions for raising the awareness of Brazilian dentists with regard to the evaluation of immunization status for this disease.

Dentists who adequately employ IPE, those who obtain a patient history regarding hepatitis B, those with a family history of hepatitis B and those more recently graduated had a greater frequency of self-reported immunization for hepatitis B. As mentioned above, dentists with a more favourable behaviour with regard to infection control measures are more apt to adhere to an immunization evaluation. Living with the disease in the family setting may have sensitized these dentists to adhering to every prevention protocol in the professional setting. Likewise, dentist who more recently graduated may have been submitted to a different training/education process in relation to infection control measures. Further education may be appropriate in order to impress upon dental students and dentists alike the importance of knowledge on their own vaccination and immunization status.

The present study has some limitations that must be recognized. Cross-sectional studies are carried out either at a single point in time or over a short period. Thus, associations identified in cross-sectional studies should not be considered a causal relationship. However, there is a lack of studies that concomitantly assess the knowledge of dentists regarding their own hepatitis vaccination and immunization status, as we performed in the present study.

Given the huge burden of hepatitis B infection worldwide and the number of advances made in the past several decades, it is surprising that more progress in limiting the infection has not been made. Hepatitis B continues to spread in endemic areas where universal vaccination has not yet been achieved. The availability of the vaccine and its use in preventing neonatal transmission as well as the increasing use of suppressive therapies should yield greater gains in the eradication of hepatitis B in upcoming generations [3,4]. Moreover, dentists should be better instructed with regard to the importance of compliance to the vaccination and taking the post-vaccination test in order to assist in the elimination of hepatitis B.

## Conclusions

The prevalence of hepatitis B vaccination among Brazilian dentists was associated to gender, blood transfusion and risk behaviours. A low percentage of dentists reported having taken the post-vaccination test and few were aware of their immunization status. We strongly recommend that dentists, as a potential risk group, should know their immunization status so that those who require revaccination can obtain it.

## Competing interests

The authors declare that they have no competing interests.

## Authors' contributions

VLSR, MHGA, SMP, RT and IAP conceptualized the rationale and design of the study. VLSR and MHGA performed the statistical analysis and interpretation of the data. VLSR, MHGA, SMP and IAP drafted the manuscript. All authors read and approved the final manuscript.
